# Movement Sonification in Stroke Rehabilitation

**DOI:** 10.3389/fneur.2018.00389

**Published:** 2018-06-01

**Authors:** Gerd Schmitz, Jeannine Bergmann, Alfred O. Effenberg, Carmen Krewer, Tong-Hun Hwang, Friedemann Müller

**Affiliations:** ^1^Institute of Sports Science, Leibniz University Hannover, Hannover, Germany; ^2^Schön Klinik Bad Aibling, Bad Aibling, Germany; ^3^German Center for Vertigo and Balance Disorders, Ludwig-Maximilians University of Munich, Munich, Germany; ^4^Department of Sport and Health Sciences, Technical University Munich, Human Movement Science, Munich, Germany; ^5^Institute of Microelectronic Systems, Leibniz University Hannover, Hannover, Germany

**Keywords:** movement sonification, motor rehabilitation, stroke rehabilitation, arm movements, acoustic feedback

## Abstract

Stroke often affects arm functions and thus impairs patients' daily activities. Recently, several studies have shown that additional movement acoustics can enhance motor perception and motor control. Therefore, a new method has been developed that allows providing auditory feedback about arm movement trajectories in real-time for motor rehabilitation after stroke. The present article describes the study protocol for a randomized, controlled, examiner, and patient blinded superiority trial (German Clinical Trials Register, www.drks.de, DRKS00011419), in which the method will be applied to 13 subacute stroke patients with hemiparesis during 12 sessions of 30 min each as additional feedback during the regular movement therapy. As primary outcome, a significant pre-post-change in the Box and Block Test is expected that exceeds the performance increase of 13 patients who will be provided with sham-acoustics. Possible limitations of the method as well as the study design are discussed.

## Introduction

### Background

Stroke is the second most common cause of death among the neurological disorders. The great majority of patients who survive a stroke have to rely on health care support afterwards (1). Sensory and motor impairments can lead to dramatic limitations of everyday motor skills and temporary or permanent disability. Most often arm functions are impaired and hamper patients during activities of daily living (2). Hemiparesis, for example, affects spatial and temporal arm motor control and results in disturbed movement trajectories, lower movement amplitudes and enhanced movement times (3). Therefore, one important goal of motor rehabilitation is the improvement of arm functions. Some therapies like the Arm Ability Training (4) or the Constraint Induced Movement Therapy (5) predominantly focus on the improvement of the motor components of the arm movement system. However, Bastian points out that efficacy of stroke rehabilitation might be improved by methods that combine perceptual- and motor oriented approaches (6). A recent study with healthy participants showed a higher efficacy of a sensorimotor compared to a purely motor orientated approach, accordingly, although both approaches address the same adaptation mechanisms (7). An example for a perception-oriented approach for stroke rehabilitation is Ramachandran's mirror visual feedback method. It seems to reestablish congruency between motor commands and visual feedback in patients that watch a mirror image of the unimpaired arm during bilateral movements. Some of these patients report not only to see the impaired arm, but also to feel it moving. A probable explanation is that mirror visual feedback revives temporarily inactive motor neurons and/or ipsilateral corticospinal pathways (8).

As alternative to vision-oriented approaches, a specific feature of recently developed methods is the implementation of auditory signals and sounds to generate additional perceptual information about movement quantities and qualities (9, 10). In particular, music has been shown to be an efficient add-on in stroke therapy: Schneider et al. (11) showed that a music based arm therapy can outperform highly established approaches like the constraint induced movement therapy. Chen et al. (12) reported from a proof of concept case study on five stroke patients that rhythmic auditory cueing enhanced movement speed. Furthermore, two-state continuous musical feedback increased elbow extension as well as shoulder flexion and reduced compensatory trunk movements. Growing evidence suggests that music-supported therapy is superior to conventional physiotherapy without music, probably because it acts on multiple levels and addresses motor, cognitive, and emotional mechanisms (13).

Furthermore, some studies indicate beneficial effects of continuous auditory feedback for movement rehabilitation after stroke. For example, Maulucci and Eckhouse (14) reported that stroke patients relearned functional movement paths faster when they were provided with auditory feedback about spatial deviations from reach paths performed by healthy persons. Secoli et al. (15) found that auditory feedback improved performance in a movement tracking task performed during robot-assisted arm training in patients with chronic left hemiparesis. However, other results were equivocal: According to Robertson et al. (16), feedback about hand orientation during reaching seems to be beneficial for patients with right hemisphere lesions, but detrimental for patients with left hemisphere lesions. Based on a systematic literature review, Molier and colleagues see a possible benefit of performance feedback and augmented auditory feedback, although the determinants for their efficacy remain largely unknown (17).

Since stroke often impairs somatosensation (18, 19), recovery of arm functions might benefit from methods that support proprioception, particularly. Hereto, Sihvonen et al. (13) argue that music-supported therapy might be effective, again, because patients generate an internal expectation about when the next note is going to be heard and thereby improve their movement timing. However, by considering proprioception as integrated percept of multiple sensory streams from multiple receptors which is experienced as motion and position sense, further methods might address specific proprioceptive mechanisms and thereby support the relearning of functional movement patterns after stroke. The method of movement sonification might have this potential. Movement sonification represents a concept for mapping movement parameters to sound in order to create novel perceptual streams congruent to the time course of kinematic or dynamic movement parameters (20). This method differs conceptually from providing feedback on performance errors, because it allows to design artificial perceptual streams structurally equivalent to perceptual streams from other modalities. It has been shown that the amendment of visual motion information by movement acoustics amplifies the activity of multimodal integration areas in the brains of observers and furthermore, activates the basal-ganglia-fronto-cortical motor loop (21, 22). Accordingly, movement sonification has been shown to support learning (23, 24) and adaptation (25) of fine motor skills, (re)learning of arm joint coordination patterns (26) and acquisition of gross motor skills (27, 28) in healthy persons. In deafferented patients, it can substitute proprioception (29). Studies on immediate effects of movement sonification on movement pattern recognition, movement synchronization and own-other discrimination (30–33) indicate that movement sonification unfolds its potentials on perception and action by linking to internal movement representations.

### Theoretical approach

The present approach is based on a further development of a method presented in Vinken et al. (31) and Schmitz et al. (34). It differs from other approaches as it focuses on sensorimotor representations of hand and arm movements as suggested by Bastian (6) for arm training in stroke rehabilitation. Studies indicate that hand and arm movements are represented in body-centered reference frames and that arm trajectories are realized on the basis of muscle synergies (35–38). Findings from Overduin and colleagues indicate that muscle synergies are represented in the brain in a time-invariant spatial as well as a time-varying spatiotemporal manner (39). d'Avella et al. (40) showed that different muscle synergies are active during movements to different directions, but a few synergies can sufficiently explain coordinated muscular activity during movements with different amplitudes, loads, forearm postures, as well as movement sequences. Such results indicate that synergies serve the implementation of a few global movement features like movement direction and amplitude which are coded by independent neuronal populations in the brain (35). Accordingly, it seems to be reasonable to design feedback related to movement direction and amplitude in an egocentric reference frame to address arm movement control and muscle synergies. Since stroke seems to disrupt muscle synergy patterns of the impaired arm (41), significant effects of such feedback might be expected for the rehabilitation of arm functions. Moreover, muscle synergy patterns are highly correlated between arms and the reorganization of muscle synergy patterns is part of the recovery process after stroke (41). By providing homogenous feedback on movements of each arm, the unimpaired arm can serve as individualized movement model as well as auditory mirror image and might support the reorganization process.

The goal of the intended clinical trial is to prove the impact of a novel method for arm movement sonification in motor rehabilitation after stroke. The method provides real-time feedback about three-dimensional wrist movements in relation to the trunk. Auditory feedback informs about the angular direction of movements in the horizontal and vertical plane, the radial amplitude as well as the absolute velocity of the wrist. Accordingly, each movement produces an unequivocal sound which represents additional sensory feedback. We hypothesize that patients benefit from the real time feedback during movements with the impaired arm due to the structural equivalence of the sound to movement information from other modalities which can amplify activity of multisensory integration areas in the brain, support motor control and substitute partially lost proprioception as indicated by Scheef et al. (21), Schmitz et al. (22), Effenberg et al. (28) and Danna and Velay (29). A pilot study with a precursor version of this method provided encouraging results as four stroke patients showed improved performance in the Box and Block test after five training sessions (34). Furthermore, a related method has recently been applied in a randomized controlled clinical trial by Scholz et al. (42). After an exploration-phase, stroke patients learned to play simple melodies by moving their impaired arm in 3D-Cartesian space. Ten training sessions of 20 min each with this musical sonification reduced pain according to the pain-score of the Fugl-Meyer test, enhanced hand functions as assessed by the Stroke Impact Scale and increased smoothness of reaching as shown by kinematic analyses. Effect sizes were moderate. The present approach differs from the method presented in Scholz et al. (42) mainly by the way arm movements are sonified and by capturing the whole upper body. This allows providing intuitive feedback for both arms while controlling for upper body movements. Thus, the present method prospects even larger effects.

### Objectives

The primary objective of this trial is to investigate the effectiveness of the above mentioned approach for real-time movement sonification on motor abilities of the paretic upper limb in subacute stroke patients. The hypotheses are that, compared to patients provided with an auditory control stimulus, patients provided with real-time movement sonification (1) improve gross motor dexterity of the paretic upper limb assessed with the Box and Block test, and (2) motor function of the paretic arm and hand measured with the Action Research Arm Test and the Stroke Upper Limbs Capacity Scale.

### Trial design

The present trial is designed as a randomized, controlled, assessor and patient blinded superiority trial with two parallel groups. Randomization is performed as block randomization with 1:1 allocation.

## Methods

### Study setting and eligibility criteria

All subjects included in the study are inpatients at a rehabilitation hospital in Germany. They meet the following inclusion criteria: hemiparesis of the upper extremity (SULCS score ≥3) after a unilateral ischemic or hemorrhagic stroke (4 weeks to 6 months after stroke onset), the functional ability to pick up a wooden cube (2.5 cm in size) with the paretic hand, and age between 18 and 80 years. Patients with unstable fracture, the inability to sit for 30 min, or severe aphasia or cognitive impairment, which compromises the implementation of the assessments or the therapy are excluded from the trial.

### Interventions

Subjects enrolled in this study are randomized in equal proportions between sonification and sham-acoustics, receiving either training for the upper extremities with real-time movement sonification (intervention group) or training with sham-acoustics (control group). During the intervention phase, subjects of both groups receive movement therapy for the upper extremities at 4 days per week for 3 weeks, i.e., a total of 12 sessions. A therapy session takes 30 min. Within each study therapy session, gross motor arm movements are performed focusing on (a) reaching, (b) grasping, (c) bimanual activities, and (d) velocity. Exercises belonging to those categories are performed in blocks of 5 min. An exercise catalog can be used by the therapists containing ideas for arm movements of each category. Content and repetitions are recorded for later analysis. A break and a short calibration of the XSens system is scheduled between the 5-min blocks. Subjects of both groups wear the sonification system (straps, sensors, head phones, on-body controller) during the therapy.

Once a patient is enrolled in the study, the study site makes every reasonable effort to follow the patient for the entire study period. If study sessions are canceled due to indisposition of the patient or the therapist, or due to technical issues, one additional session per week can be scheduled. The intervention period should not exceed 3 weeks. Adherence to therapy is monitored by documenting therapy failures, therapy durations, and drop-outs.

The following individual criteria were defined for discontinuing the allocated intervention: incidence of a new disease or complication of the underlying disease, which makes continuation of the study impossible, and at the patients' request or at request of the legal representative. Patients who discontinue the intervention are considered *off intervention* and follow the same schedule of measurements as patients who finished the intervention. Discontinuation of the intervention is not a reason for withdrawal from the study. Patients are free to withdraw from the study for any reason at any time. The investigator also may withdraw patients from the study to protect their safety or if they are unwilling and unable to comply with the study procedures.

All patients included in the study are inpatients at the rehabilitation hospital and receive the normal therapy setting during the study period. The only intervention which is prohibited during the intervention period is robot-assisted training for the upper extremities.

### Arm motion tracking and sonification

Arm movements are tracked with a mobile motion capture system (MTx miniature 3DOF inertial orientation tracker; Xsens Technologies BV, Enschede, The Netherlands). It contains seven inertial sensor units, which are composed of three accelerometers, three gyroscopes, and magnetic sensors allowing measuring three dimensional orientation. All sensors are connected by cable with an on-body controller (XBus Master) worn at a belt which transmits synchronized sensor data (50 Hz) wirelessly to a laptop (Bluetooth protocol 2,400–2,500 MHz). Sensors are fixated by velcro straps and aligned to seven body parts representing a kinematic chain (sternum, shoulders, upper and lower arms). By comparing orientation of two interconnected sensors and considering predefined segments' lengths it becomes possible to determine joint angles and calculate relative joint positions based on forward kinematics (43), here the relative wrist position in relation to the intersection of shoulder axis and spine of a biomechanical upper body model.

**Figure 1:**
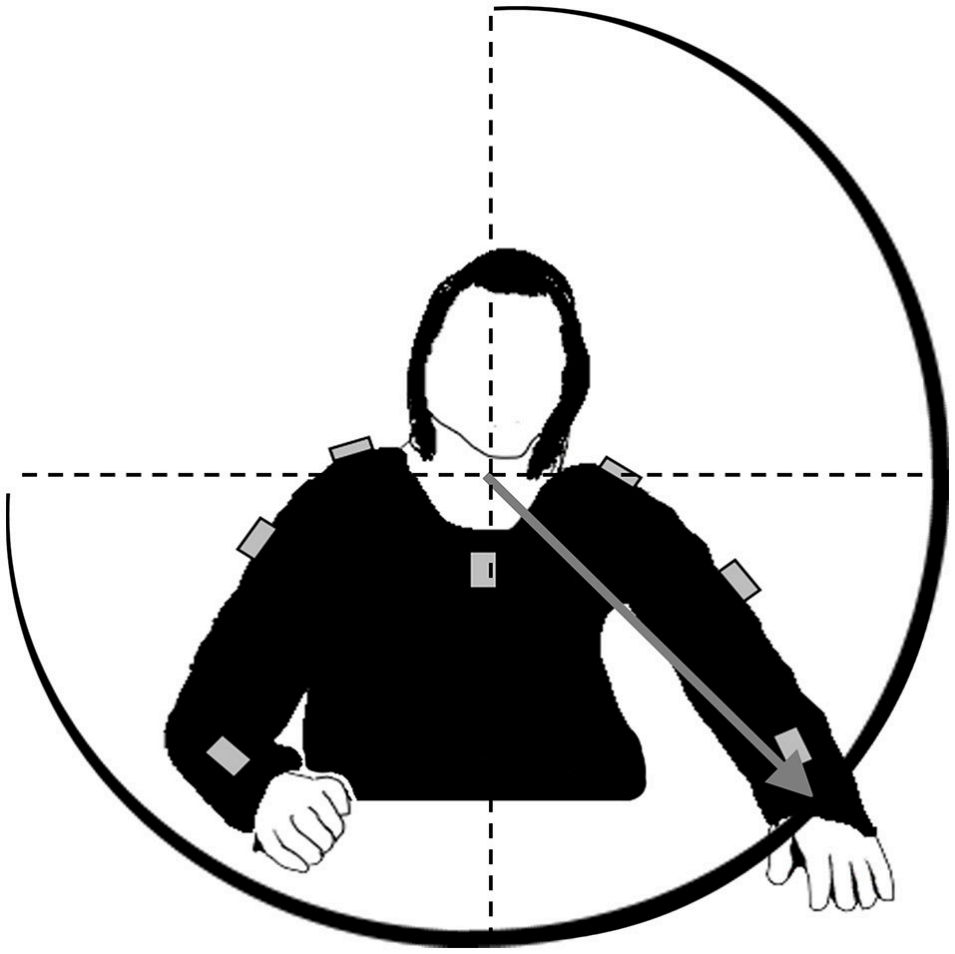
Loci of the sensors (gray boxes) at patients' upper body. Sensor data are fused to calculate spherical coordinates of the wrist in a reference frame with origin at the upper body, i.e., the intersection between spine and shoulder axis. Four parameters are mapped onto sound: the angle between gray vector and sagittal plane (azimuth angle), the angle between vector and horizontal plane (elevation angle), vector length (radial amplitude), and absolute velocity of the wrist.

Wrist position is calculated in spherical coordinates, i.e., each posture is determined by the azimuth angle, the elevation angle as well as the radial distance between wrist and origin of the spherical coordinate system (Figure 1). These data are submitted to the open source software applications PureData and CSound for sonification. The sonification concept is inspired by ecological relationships between sound and energy like it is given for the sound amplitude, which is usually determined by the amount of energy being transformed by the sound-emitting event: The harder a tennis player hits the ball with the racket, the louder the impact boom will sound (20). Such ecological relations are well established within the hearing system and the perceptual generation of such kind of auditory information does not need conscious attention. The sonification technique is based on frequency modulation of a synthesized sound with a sawtooth wave form. The carrier frequency, which is the basic frequency, is set to 200 Hz for the left arm and to 300 Hz for the right arm when the arms are hold in a neutral position besides the body (elevation angle 0°). Arm elevation increases sound frequency by a maximum of 200 Hz, which is achieved when both arms are stretched above the head (elevation angle 180°). The azimuth angle determines the panning (equal power panning) and thus the interaural intensity difference. Radial amplitude modifies the perceived brightness of the sound by a logarithmic change of the frequency regulation index between 0 and 0.15. Finally, the absolute velocity of the wrist defines the sound amplitude and thus the loudness. Higher velocities result in higher sound amplitudes. Thus, right and left arm movements produce and modify one sound each which are provided to the patients of the experimental group wirelessly via headphones. Notably, no sound can be heard when the arms are at rest. The control group is provided with sham-acoustics. Arm movements produce the sound of ocean waves, which are not altered by the movement trajectory.

The volume of the movement sonification and the sham-acoustics is adapted according to the patients' preference (maximal 65 decibel).

### Outcomes

Primary outcome measure is the Box and Block Test (44). The test is a measure of unilateral gross manual dexterity. It counts the number of wooden blocks that can be transported from one compartment of a box to another compartment within 1 min. The Box and Block test shows high test-retest and interrater reliabilities in elderly subjects and subjects with neurological disorders (45, 46). The construct validity of the test is high when compared with the ARAT and the Fugl-Meyer test (45, 46). The Box and Block test is suitable to detect changes over time in stroke patients (47).

Secondary outcome measures are the Action Research Arm Test (ARAT) and the Stroke Upper Limbs Capacity Scale (SULCS). The ARAT assesses mainly the ability to handle smaller and larger objects with a variety of qualitatively rated items. It includes four subtests: grasp (6 items), grip (4 items), pinch (6 items), and gross movement (3 items). The scores for each item range from 0 to 3. We use the standardized protocol published by Yozbatiran et al. (48) to assess the ARAT. The test shows high intrarater reliability and interrater reliability. Validity is high when compared to the Fugl-Meyer test and it is sensitive to detect changes (47).

The SULCS assesses the capacity of the paretic upper limb in stroke patients. It consists of 10 items which represent tasks that are related to daily activities (49). The items assess proximal arm capacity without need for active wrist or finger movement (3 items), arm capacity combined with basic hand capacity (grasp tasks without manipulating, 4 items), and advanced hand capacity (manipulating tasks, 3 items). The scale has good interrater reliability and a high construct validity when compared with the ARAT and the Rivermead Motor Assessment (RMA) (50).

All these outcome measures are assessed at baseline before the start of the intervention, at post-test after the last intervention, and at follow-up test 2 weeks after the end of the intervention. The measures are assessed by an assessor blind to treatment allocation. The assessor is experienced and trained in performing the clinical assessments.

Differences between baseline and post measurement, and between baseline and follow-up measurement will be calculated for all outcome measures to determine short-term and long-term changes. These changes will be compared between groups. The primary and secondary outcome measures will be presented as means and standard deviations or as medians and 25th and 75th percentiles for each group.

### Participant timeline

The study timeline shown in Table 1 presents an overview of the time schedule of enrolment, interventions, and assessments of the outcome measures.

**Table 1:** Time schedule of enrolment, interventions, and assessments.

	**Enrolment**	**Allocation**	**Post-allocation**		
**TIMEPOINT**		**0**	**Baseline**	**Post**	**Follow-up**
ENROLMENT:					
Pre-screening	X				
Eligibility screening	X				
Informed consent	X				
Allocation		X			
Randomization		X			
INTERVENTIONS:					
Movement sonification			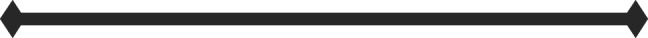		
Sham-acoustics			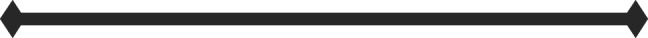		
ASSESSMENTS:					
Box and Block Test			X	X	X
Action Research Arm Test			X	X	X
Stroke Upper Limbs Capacity Scale			X	X	X

### Sample size

The sample size calculation for this trial is based on the pilot study by Schmitz et al. (34) investigating the feasibility of movement sonification in stroke patients. The study found a small, but statistically significant effect and a high correlation between the number of blocks which were transported in the Box and Block test before and after five 20-min sessions with movement sonification. As the intervention period is much longer in this trial, we assume a medium effect. For an effect size of *f* = 0.2, a correlation among repeated measures of 0.7, a power of 80%, and a significance level of α = 0.05 a total sample size of 26 subjects is required. The sample size calculation was performed using G^*^Power.

A dropout rate of 20% was anticipated, consequently a minimum number of 32 subjects has to be enrolled in the study.

### Recruitment

In the pre-screening, a scientific staff member determines on a daily basis all stroke patients admitted to the hospital. These patients are screened for eligibility by the study coordinator. Patients who fulfill all inclusion and exclusion criteria are approached with the study information. If the patient is interested in the study and agrees to participate, written informed consent is obtained. If the patient has a legal representative, the study information is also provided to the legal representative and he gives written informed consent. Patients who are not yet, but potentially may become eligible, are followed by the study coordinator until they meet all the eligibility criteria.

### Allocation

Patients included in the study are randomly assigned to either the control or the experimental group with a 1:1 allocation as per a computer generated randomization schedule stratified by age (<60 and ≥60 years) and lesion side (left and right sided) using blocks of random sizes. The block sizes will not be disclosed to ensure concealment. The randomization schedule will be concealed until the primary endpoint will be analyzed. The allocation is done by a scientific staff member not directly involved in the project. The staff member sends a form with the allocated intervention to the therapist who is not involved in assessing the outcome measures.

### Blinding

The information about treatment allocation is not given to the patient in order to ensure blinding as long as possible. However, due to the nature of the intervention, blinding of the patient during the intervention may be difficult. Blinding of the therapist is not possible.

### Data management

Data is collected by a blinded assessor using data based case report forms. All data are entered into an electronic database by a scientific staff member at the study site who is not involved in data collection. Original data forms will be kept on file at the study site in locked cabinets. Access to the study data will be restricted to authorized staff members. Incremental back-ups of the electronic database will be performed on a daily basis. The database is protected by a password.

After termination of the study and the data verification, all files will be archived for a period of 10 years.

### Statistical methods

Descriptive statistics including means and standard deviations or medians and 25th and 75th percentiles for continuous data, and frequencies for categorical data will be determined. The appropriateness of the randomization will be examined by testing for between group differences in demographical and clinical variables (e.g., age, time since stroke, SULCS score).

Among the cases available for analyses, intention-to-treat analyses will be performed. For all outcome measures, the within-subject differences between the baseline and post-test, and the baseline and follow-up test are of central interest in the intervention group compared to the control group. For the primary outcome measure, a repeated measures analysis of variance will be used. Pairwise comparisons will be generated using Tukey's method. A subgroup analysis will be performed to investigate the influence of lesion side. For the secondary ordinal outcome measures, non-parametric statistics will be used. Between-group comparisons (intervention vs. control group) will be performed to compare the short-term changes (baseline—post-test) and long-term changes (baseline—follow-up) between groups using Mann-Whitney *U*-tests. In addition within-group comparisons will be performed using Friedman tests. If the Friedman test showed significant differences, Wilcoxon matched-pairs tests will be used to compare baseline and post, and baseline and follow-up measures. Effect sizes (r) of changes between groups and within-groups will be calculated.

In addition, a per protocol analysis will be done, excluding patients who deviated from the protocol. Missing data will be replaced by the last value carried forward method.

### Data monitoring

No external monitoring of the trial procedures or data collection processes will occur and no auditing is planned for this trial.

No interim statistical analyses are planned. The study will be stopped if risks emerge which were not known before.

### Harms

In this trial, an adverse event is defined as any untoward medical occurrence in a subject without regard to the possibility of causal relationship. Adverse events will be collected and reported after the patient or his/her legal representative has provided written informed consent and the patients is enrolled in the study until follow-up test. All adverse events are evaluated with regard to the anticipation and severity of the adverse event, and the causal relation to the study intervention or study procedure.

An adverse event which occurs after enrolment but before the intervention is started, will be reported as not related to the study intervention.

An adverse event that meets the criteria for a serious adverse event between study enrolment and follow-up test will be reported to the German Federal Institute for Drugs and Medical Devices (BfArM).

### Research ethics approval

This trial is performed according to the World Medical Association Declaration of Helsinki as well as the guidelines for good scientific practice of the German Research Foundation and of the University of Hannover. It has been approved by the Ethics Committee of the Bavarian State Chamber of Physicians.

### Protocol amendments

Any modifications in the study protocol will be reported to the relevant Ethics Committee and the registration in the German Clinical Trials Register will be amended.

### Consent

The study coordinator introduces the trial to patients who fulfill all the eligibility criteria of the study. The patients also receive an information sheet about the study (informed consent form) and the study coordinator discusses the trial with the patients in light of the information provided. Patients are then able to have an informed discussion with the principle investigator and ask questions. At least 24 h after the informed discussion, the principle investigator obtains written consent from patients willing to participate in the trial. The informed consent involves a confirmation that the patient understands the research and an assurance that their agreement to participate is voluntary. If a patient has a legal representative the informed consent form will also be provided to the legal representative. The legal representative will also have a informed discussion with the principle investigator and gives written informed consent if he agrees with study participation.

### Confidentiality

All administrative and data collection forms are identified by a coded ID number only to maintain patient confidentiality. All records that contain names or other personal identifiers, such as informed consent forms, will be stored separately from study records identified by code number. All study-related information will be stored securely at the study site. Access is limited to the staff involved in quality control and data analysis. The electronic database is password-protected. Data which will be transmitted to co-investigators of the University Hannover for analysis do not include personal identifiers.

### Access to data

Authorized research staff at the Schön Klinik Bad Aibling will have direct access to the data sets. Project team members at the University Hannover will have access by request. To ensure confidentiality, data dispersed to project team members will be blinded of any identifying participant information.

### Ancillary and post-trial care

All participants are inpatients at the rehabilitation hospital. After completion of the study, all patients receive rehabilitation treatments and therapy according to their functional level. No specific post-trial care is planned.

The study site has an insurance to cover for harms associated with the trial. This includes cover for additional health care, compensation, or damages.

### Dissemination policy

Results of this trial will be disseminated through presentations at scientific conferences and peer-reviewed publications.

## Discussion

Stroke patients often show an impaired spatial and temporal arm control which results in disturbed movement trajectories. Movement sonification is a novel approach to map movement trajectories to sound and provide the patient with real-time auditory feedback. We hypothesize that these method might support the relearning of functional movement patterns after stroke. The goal of this clinical trial is to scrutinize the efficacy of a recently developed method for real-time movement sonification on motor abilities of the paretic upper limb in subacute stroke patients. In addition, it assesses adherence to therapy and adverse events.

The combination of perceptual and motor oriented approaches seems effective to improve motor rehabilitation after stroke (6). While visual feedback training is widely-used, acoustic feedback methods are much less prevalent and insufficiently investigated. The method of movement sonification which is applied in this trial provides the patient with additional auditory feedback about three-dimensional wrist movements in relation to the trunk during regular movement therapy. This perception-oriented approach links to internal movement representations and might address specific proprioceptive mechanisms and support relearning of functional movement patterns. Despite its potential benefits, the method has several possible limitations which have to be discussed as they might influence the study outcome.

The mobile sonification system allows to sonify up to 16 from several hundred movement parameters, concurrently. Therefore, it is highly adaptable to different movement categories. Unfortunately, applicability in arm motor rehabilitation is limited to gross-motor functions, because the motion capture is based on inertial sensor units that do not allow capturing finger or grasping movements. A system for the sonification of hand- and finger movements to support grasping actions and fine motor skills could be developed in future on the basis of data gloves with an adapted kinematic-auditory framework as the one used in the intended study.

A second limitation results from the calibration procedure in which orientations of sensor units are aligned to orientations of body limbs. Repeated recalibration is necessary, because inertial sensor data tend to drift. Thereto, patients have to take up a pre-defined pose, in which both arms are stretched. Although the pose is standardized and patients are supported by the therapist, inaccuracies have to be expected that induce noise in the kinematic-acoustic mapping. Such noise might reduce the impact of the auditory movement information during the multisensory fusion process with information from other sensory modalities (51). Although a higher accuracy might be achieved with optical motion capture systems, it was decided to base motion capture on inertial sensor units to maintain mobility as well as time efficient motion data processing to minimize latency of auditory feedback.

A third possible limitation concerns the necessity to standardize the kinematic-acoustic mapping inter-individually in the clinical study. It might be argued that a higher efficiency of the method will be achieved by adapting the kinematic-acoustic mapping to each patient individually, since impairments vary inter-individually. But by sonifying spherical coordinates (angles and standardized amplitudes), inter-individual differences seem to play a minor role and the need to adapt the kinematic-acoustic mapping diminishes. However, during the 3 weeks of movement therapy, many different arm movement have to be practiced (uni- as well as bilateral movements, different velocities, cyclic/acyclic etc.), and different movement types might require feedback on different movement parameters to achieve highest efficiency. Thereto, an adapted mapping-strategy might be beneficial.

One major study limitation of the study protocol is the blinding of the patients. Due to ethical reasons, patients are informed in the information sheet and the informed discussion that they will be randomly allocated to one of two treatment groups. They are told that the control group is provided with sham-acoustics which is not related to arm movements. After enrolment in the study, the information about treatment allocation is not given to the patient. However, some patients might notice whether they train with movement sonification or sham-acoustics. As knowledge of group allocation might influence the study outcome, it will be documented if a patient mentions awareness of his treatment group.

The study investigates the effectiveness of movement sonification in patients in the subacute phase after stroke. Further work should determine its effects in acute or chronic stroke patients.

## Author contributions

GS and JB drafted the manuscript. AOE, CK, T-HH, and FM revised it critically for important intellectual content. AOE developed the sonification. GS and AOE developed the framework for the arm sonification. T-HH contributed to the software-application and sound-synthesis. CK conceived, and JB designed the study. CK, JB, AOE, GS, and FM participated in the development of the intervention. All authors read and approved the version of the submitted manuscript.

### Conflict of interest statement

The authors declare that the research was conducted in the absence of any commercial or financial relationships that could be construed as a potential conflict of interest.
